# Nationwide Survey of Pediatric Inpatients With Hand, Foot, and Mouth Disease, Herpangina, and Associated Complications During an Epidemic Period in Japan: Estimated Number of Hospitalized Patients and Factors Associated With Severe Cases

**DOI:** 10.2188/jea.JE20180060

**Published:** 2019-09-05

**Authors:** Maria Takechi, Wakaba Fukushima, Takashi Nakano, Miki Inui, Satoko Ohfuji, Tetsuo Kase, Kazuya Ito, Kyoko Kondo, Akiko Maeda, Hiroyuki Shimizu, Yoshio Hirota

**Affiliations:** 1 Department of Public Health, Osaka City University Graduate School of Medicine, Osaka, Japan; 2 Research Center for Infectious Disease Sciences, Osaka City University Graduate School of Medicine, Osaka, Japan; 3 Department of Pediatrics, Kawasaki Medical School, Okayama, Japan; 4 Osaka City University Hospital, Osaka, Japan; 5 Department of Virology II, National Institute of Infectious Diseases, Tokyo, Japan

**Keywords:** hand foot and mouth disease, enterovirus 71, nationwide survey, pediatric hospitalization, Japan

## Abstract

**Background:**

Severe pediatric cases of hand, foot, and mouth disease (HFMD), herpangina (HA), and associated complications caused by enterovirus 71 (EV71) infection have brought substantial public health impact in Asia. This study aimed to elucidate the epidemiology of these pediatric cases in Japan.

**Methods:**

A nationwide survey was conducted using stratified random sampling of hospital pediatric departments. We estimated the number of inpatients with HFMD, HA, and associated complications between April 1 and September 30, 2010, during which EV71 was circulating predominantly. Factors associated with severe cases with ≥7 days of admission, sequelae, or outcome of death were analyzed using multivariate logistic regression.

**Results:**

During the 6-month epidemic period, the number of pediatric inpatients aged <15 years was about 2,900 (estimated cumulative incidence of hospitalized cases: 17.0 per 100,000 population). Severe cases were significantly associated with younger age. Compared to patients ≥5 years of age, the odds ratios (ORs) for <1 year of age and 1 to <3 years of age were 5.74 (95% confidence interval [CI], 2.14–15.4) and 2.94 (95% CI, 1.02–8.51), respectively. Elevated ORs for hyperglycemia (plasma glucose level of ≥8.3 mmol/L) on admission (OR 3.60; 95% CI, 0.94–13.8) were also observed.

**Conclusions:**

Disease burden of pediatric inpatients with HFMD, HA, and associated complications in Japan was described for the first time. During an EV71 epidemic, younger age and, suggestively, hyperglycemia may have been critical factors requiring more careful treatment.

## INTRODUCTION

Hand, foot, and mouth disease (HFMD) and herpangina (HA) are infectious diseases caused by non-polio enteroviruses, including coxsackievirus A (CA)6, CA10, CA16, and enterovirus 71 (EV71). Although skin symptoms, including papulovesicular and/or maculopapular rash on palms, soles, sometimes knees and elbows, and vesicles/ulcers in the mouth, are known to be typical clinical manifestations of HFMD and HA, severe complications, such as central nervous system (CNS) involvement with or without typical skin symptoms, sometimes develop. The outbreak of these diseases occurs mainly among infants throughout the year in tropic/subtropic countries and during the summer in Japan.

Since the late 1990’s, outbreaks of HFMD and HA cases, including CNS complications regardless of typical skin symptoms, mainly caused by EV71 infection, have occurred in the Western Pacific Region.^1^^–^^11^ In these outbreaks, cases involving encephalitis, brainstem encephalitis, and acute flaccid paralysis were reported, and a number of children died,^2^^,^^3^^,^^12^^–^^14^ resulting in a substantial public health impact. Subsequently, special attention has been given to EV71 as a pathogen that can have CNS involvement in HFMD and HA cases.

In Japan, HFMD and HA are classified as Category V notifiable infectious diseases under the National Epidemiological Surveillance of Infectious Diseases (NESID), and the pediatric sentinel medical institutions report the number of cases weekly. Approximately 10% of pediatric sentinel medical institutions for the NESID also participate in the Infections Agent Surveillance (IAS) as sentinels.^15^ In 1997, 2000, and 2003, there were major epidemics of HFMD, and the dominant serotype of enteroviruses isolated from HFMD patients was EV71.^16^^,^^17^ Smaller epidemics of HFMD continued until 2006, when EV71 and CA16 were mainly isolated from patients.^18^ In 2010, Japan again experienced a large epidemic of HFMD, and EV71 was the predominant pathogen in patients (634/959; 66%) according to monthly reports issued by the IAS.^19^ Thereafter, large-scale HFMD outbreaks have occurred every 2 years (in 2011, 2013, 2015, and 2017) in Japan; however, the major causative agent of the outbreaks was CA6 instead of EV71.^20^ While such surveillance systems are available, few studies have investigated epidemiological characteristics of or factors associated with severe cases of HFMD, HA, or enterovirus infection.

We conducted a nationwide survey on pediatric inpatients with HFMD, HA, and associated complications during an epidemic period in 2010, during which EV71 was circulating predominantly. In order to focus on patients who required hospitalization or intensive medical care, we restricted our target population to only inpatients. Our objectives were to estimate the number of pediatric inpatients with HFMD, HA, and associated complications during the epidemic period, and to explore factors associated with severe cases. Since patients not presenting with skin eruptions or rashes despite EV71 infection have been reported,^3^^,^^12^ cases of suspected enterovirus infection with no skin symptoms typical of HFMD or HA were also included in our survey.

## METHODS

### Overall design and study year

A retrospective nationwide survey was conducted using a protocol for epidemiological research on intractable (rare) diseases, which was designed by the Study Group of Epidemiological Research of Intractable Diseases Japan.^21^ This protocol was originally developed with the consideration that patients who have intractable diseases are likely to visit larger hospitals. We utilized the protocol in our study because HFMD and HA patients requiring hospitalization due to severe manifestations are likely to visit larger hospitals.

**Figure 1.**
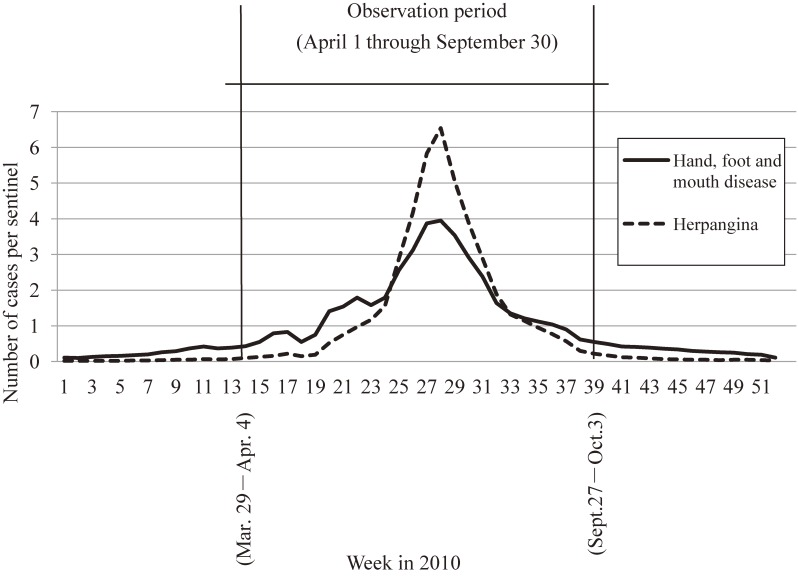
Epidemic curve showing number of reported cases per sentinel clinic per week through surveillance system in Japan in 2010

Two-stage surveys were prepared according to the protocol. The first-stage survey estimated the number of patients during the observation period, and the second-stage survey described epidemiological features of the patients.^21^^,^^22^ The observation period covered all epidemiological weeks in which at least one HFMD or HA case per sentinel was reported in the NESID (Figure 1).^23^ Given that April was the start of the administrative year in Japan, the observation period was arranged as a full 6 months (half year) beginning with April and continuing through the end of September.

Our study year 2010 was characterized by predominant circulation of EV71.^19^ NESID data showed the number of HFMD and HA cases per sentinel as 49.87 and 45.97, respectively.^24^ According to the above-mentioned IAS, among 959 specimen samples from HFMD patients tested at municipal public health institutes across Japan, EV71 was isolated from 634 (66%) samples.^19^ EV71 was isolated vigorously in the western part of Japan.^25^ In previous years in which EV71 was also predominantly isolated, and data from NESID and IAS were available, case numbers of HFMD/HA per sentinel were 68.96/49.45 (2000), 56.78/48.89 (2003), and 33.16/38.21 (2006).^24^ In those years, proportions of EV71 isolated from specimen samples from HFMD patients were about 50% to 75%.^16^^,^^17^ In 2011, case numbers of HFMD doubled compared to 2010 (case numbers of HFMD/HA per sentinel: 110.9/44.39),^24^ but EV71 was not the predominant pathogen among HFMD patients (36/2001; 2%).^19^

### Survey targets, case definition, and data collection

**Figure 2.**
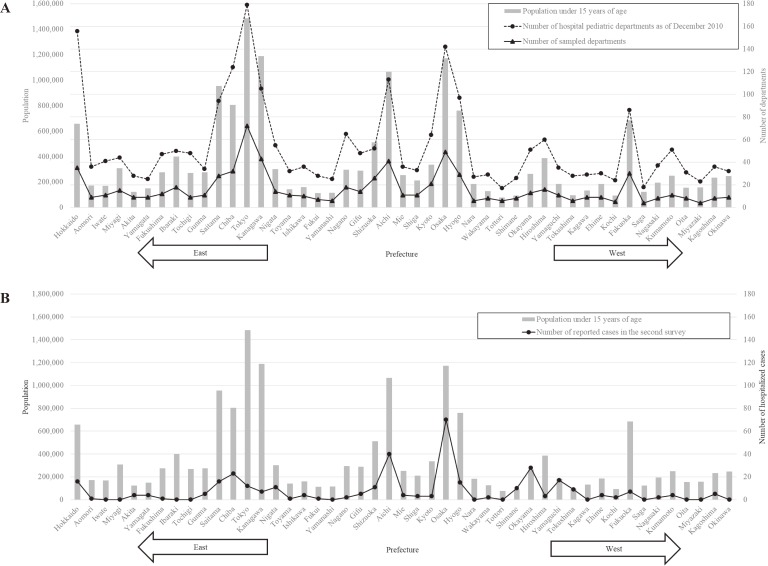
(A) Prefectural distribution of Japanese population under 15 years of age in 2010, the number of hospital pediatric departments as of December 2010 in Japan, and the number of sampled departments in this survey; (B) Prefectural distribution of Japanese population under 15 years of age in 2010 and the number of reported cases in the second survey.

Our survey targets were selected as follows: 1) all hospital pediatric departments in Japan as of December 2010 (2,507) were identified using publicly available hospital directories; 2) they were then categorized into seven survey strata according to bed capacities and characteristics of the hospitals (<100, 100–199, 200–299, 300–399, 400–499, ≥500, and university hospitals regardless of bed capacities); and 3) a fraction of 5%, 10%, 20%, 40%, 80%, 100%, and 100% were randomly sampled from respective strata. A total of 760 departments were selected as our survey targets. Prefectural distribution of 2,507 hospital pediatric departments in Japan and 760 sampled departments in this study aligned with that of the Japanese population under 15 years of age (Figure 2A).

Japanese pediatric patients who satisfied the following case definitions were to be reported: (1) met at least one definition of HFMD, HA, and eight associated complications (aseptic meningitis, brainstem encephalitis, encephalitis, encephalomyelitis, acute flaccid paralysis, autonomic nervous system dysregulation, pulmonary edema/hemorrhage, and cardiorespiratory failure) that were proposed by the World Health Organization Regional Office for the Western Pacific (WPRO) and the Regional Emerging Disease Intervention Center (Table 1, hereafter referred to as ‘WPRO definitions’)^26^; (2) were younger than 15 years of age; and (3) were hospitalized at any point of time during the observation period. In the first-stage survey, survey target departments were asked via reply-postcards to report the number of patients and, among them, the number of deaths. The first-stage survey started in January, and reminders were sent in March 2011.

**Table 1.** Clinical case definitions of hand, foot, and mouth disease; herpangina; and associated complications proposed by the World Health Organization Regional Office for the Western Pacific and the Regional Emerging Disease Intervention Center

Hand, foot, and mouth disease (HFMD):	Febrile illness with papulovesicular rash on palms and soles, with or without vesicles/ulcers in the mouth. Rash may occasionally be maculopapular without vesicular lesion, and may also involve the buttocks, knees or elbows, particularly in younger children and infants

Herpangina (HA):	Febrile illness with multiple oral ulcers on the posterior parts of the oral cavity

Associated complications	

Aseptic meningitis:	Febrile illness with headache, vomiting and meningism associated with presence of more than 5–10 white cells per cubic millimeter in cerebrospinal (CSF) fluid, and negative results on CSF bacterial culture

Brainstem encephalitis:	Myoclonus, ataxia, nystagmus, oculomotor palsies, and bulbar palsy in various combinations, with or without MRI. In resource-limited setting, the diagnosis of brainstem encephalitis can be made in children with frequent myoclonic jerks and CSF pleocytosis.

Encephalitis:	Impaired consciousness including lethargy, drowsiness or coma or seizures or myoclonus.

Encephalomyelitis:	Acute onset of hyporeflexic flaccid muscle weakness with myoclonus, ataxia, nystagmus, oculomotor palsies, and bulbar palsy in various combinations

Acute flaccid paralysis:	Acute onset of flaccid muscle weakness and lack of reflexes

Autonomic nervous system (ANS) dysregulation:	Presence of cold sweating, mottled skin, tachycardia, tachypnea, and hypertension

Pulmonary edema/hemorrhage:	Respiratory distress with tachycardia, tachypnea, rales, and pink frothy secretion that develops after ANS dysregulation, together with a chest radiograph that shows bilateral pulmonary infiltrates without cardiomegaly.

Cardiorespiratory failure:	Cardiopulmonary failure is defined by the presence of tachycardia, respiratory distress, pulmonary edema, poor peripheral perfusion requiring inotropes, pulmonary congestion on chest radiography and reduced cardiac contractility on echocardiography.

The second-stage survey was started in June 2011. Structured patient-investigation forms were sent to departments that reported one or more patients in the first-stage survey. Survey target departments were requested to complete the forms based on already existing patients’ medical records. In order to exclude patients whose illnesses might be attributed to causes other than enterovirus infection, departments were asked to not complete forms for patients meeting the following criteria: 1) pathogenic agent other than enterovirus identified or strongly suspected by attending pediatricians (eg, aseptic meningitis or encephalitis caused by mumps, herpes simplex virus, or influenza virus), or 2) judged by attending pediatricians as having other causes (eg, chronic autonomic nervous dysregulations, cardiorespiratory failure caused by Kawasaki disease, or trauma).

Survey items in the patient-investigation forms in the second-stage survey included sex, date of birth, presence of underlying medical conditions (asthma, allergy, heart disease, kidney disease, endocrine disease, neurological disease, immune suppressive condition, and other diseases), data on admission (diagnosis, presented symptoms, and results of physical and blood examinations), date of onset (based on the occurrence of rashes and/or oral ulcers, fever [≥37.5°C], and neurological symptoms), results of cerebrospinal fluid (CSF) examination, results of etiological agent identification, date of discharge, and clinical outcome (recovered, sequelae, transferred, death). Reminders were sent in August 2011. Informed consent from the patients and guardians was waived since the study collected already existing information archived in medical records. The study was approved by the ethics committee of Osaka City University Graduate School of Medicine (number registered to ethical committee: 1932).

### Estimation of the number of patients

The total number of patients in Japan during the epidemic period was estimated using the following formula: estimated total number of patients = number of cases reported/(sampling proportion × response proportion) = number of cases reported/(number of departments with response/number of departments in Japan).^27^^,^^28^ Taking into account the proportion of patients excluded in the second-stage survey, we furthermore performed the following calculation, “estimated total number of patients multiplied by (1 − proportions of such excluded cases)”^29^ and determined the corrected total number of patients. Estimated numbers of patients were calculated separately for each survey stratum, which were then summed up to obtain total number. Ninety-five percent confidence intervals (CIs) were computed according to the multinomial hypergeometric distribution under the assumption of random response.

Cumulative incidence of hospitalized cases during the 6-month epidemic period was calculated as the estimated number of patients divided by the population under 15 years of age from the 2010 national census data in Japan (*n* = 16,839,170).

### Evaluating factors associated with severe cases

Using data from the patient-investigation forms in the second-stage survey, we evaluated factors associated with severe cases. Following a precedent nationwide survey study on HFMD in Japan,^30^ severe cases were defined as those with ≥7 days of hospitalization, sequelae, or outcome of death. Patients with less than 7 days of hospitalization were defined as non-severe.

Continuous variables of patients’ characteristics or laboratory findings on admission were transformed into categorical data. Age was categorized as approximate quartile of distribution in non-severe patients (<1, 1 to <3, 3 to <5, and ≥5 years). Elevated aspartate transaminase (AST), alanine aminotransferase (ALT), and creatine kinase (CK) were defined as levels over 97.5% of the clinical standard value for pediatric patients,^31^ and elevated C-reactive protein (CRP) level was defined as ≥0.30 mg/dL. Following reports from previous investigations,^32^^,^^33^ leucocytosis was defined as a white blood cell (WBC) count of ≥17,500/mm^3^, and hyperglycemia was defined as blood glucose level of ≥8.3 mmol/L. Duration from symptom onset to admission was categorized into <3 days and ≥3 days based on a prior study that suggested delayed medical evaluation was associated with severity.^34^

A multivariate logistic regression model was used to calculate the odds ratio (OR) with 95% CI as a measure of association between independent variables and severe cases. Variables considered in the multivariate analysis were those for which *P* < 0.20 was observed in univariate analysis or those considered as medically significant or having potential associations, as suggested from previous studies. In primary multivariate analyses (model 1), sex, age, presence of associated complications, underlying medical condition, fever ≥39.0°C on admission, days of symptom onset to admission (≥3 vs <3 days), leucocytosis, and elevated ALT and CRP levels were included as independent variables. Hyperglycemia was put in an additional model (model 2), despite data missing in 23% of patients, because this factor had been strongly suspected as associated with complications in previous studies.^33^^,^^35^ Statistical significance was defined as *P* < 0.05, and *P* > 0.05 but <0.1 was considered as marginal significance. All tests were two sided. Hosmer-Lemeshow tests were used to assess goodness of fit for multivariate analysis (considered as valid when *P* > 0.05). We used SAS version 9.4 (SAS Institute, Cary, NC, USA) to perform all analyses.

## RESULTS

Table 2 shows results of the first-stage survey and estimated number of inpatients with HFMD, HA, and associated complications. Out of 760 departments sampled, 521 departments replied (response proportion, 69%). Among these, 125 departments reported one or more cases, resulting in the identification of 1,094 patients.

**Table 2.** Results of the first-stage survey and estimated number of admitted patients

Strata		Number of departments in Japan^a^	Number of departments sampled (%)	Number of responding departments (%)	Number of departments reporting ≥1 patient	Number of patients reported	Estimated number of patients	Proportion of patients excluded in the second-stage survey^b^	Corrected number of patients^c^ (95% CI)
Hospitals with beds of	<100	821	41 (5)	24 (59)	1	36	1,232	0.02	1,197 (277 to 2,117)
	100–199	538	53 (10)	31 (59)	2	23	399	0.23	307 (96 to 518)
	200–299	315	63 (20)	37 (59)	9	38	324	0.23	248 (120 to 378)
	300–399	320	128 (40)	86 (67)	17	363	1,351	0.16	1,129 (−710 to 2,969)
	400–499	188	150 (80)	109 (73)	29	183	316	0.15	270 (121 to 417)
	≥500	204	204 (100)	136 (67)	48	360	540	0.48	280 (46 to 514)
University hospitals		121	121 (100)	98 (81)	19	91	112	0.43	64 (22 to 106)

Total		2,507	760 (30)	521 (69)	125	1,094	4,273	0.33	2,859 (1,207 to 4,511)

CI, confidence interval.

^a^As of December 2010.

^b^Proportions of patients that were reported on the first-stage survey but excluded on the second-stage survey.

^c^Number of patients was corrected as “Estimated number of patients” × (1 − proportions patients excluded in the second-stage survey) for each stratum.

In the second-stage survey, from these 125 departments, 85 departments responded (response proportion: 68%) and identified 352 patients. These 85 departments originally reported 526 patients in the first-stage survey, indicating that 33% (174 patients) met the exclusion criteria. Taking this proportion into account, the number of pediatric patients who were admitted due to HFMD, HA, and associated complications during the observation period was estimated to be 2,859 (95% CI, 1,207–4,511). Accordingly, estimated cumulative incidence of hospitalized cases among children <15 years of age during the 6-month epidemic period was 17.0 per 100,000 population. Two out of 352 patients had an outcome of death (case-fatality proportion: 0.57%). The largest number of cases (*n* = 70) was reported from Osaka Prefecture, which had the third highest population under 15 years of age (*n* = 1,172,291) in Japan. Subsequently, 40 and 28 cases were reported from Aichi Prefecture and Okayama Prefecture, respectively. Altogether, larger numbers of patients were reported from western parts of Japan than from eastern parts (Figure 2B).

Among 352 patients identified from the second-stage survey, information on outcome measure was available for 343 patients, of which 285 were non-severe cases and 58 were severe cases (Table 3). One hundred and ninety-five patients (57%) were male. There were 156 patients (45%) with associated complications. One patient with encephalomyelitis presented with acute disseminated encephalomyelitis (ADEM) occurring 3 weeks after HFMD onset. Patients with sequela were a 3-year-old girl without HFMD/HA and a 4-year-old boy with HFMD, both complicated by brainstem encephalitis. For the two patients with outcome of death, a 2-year-old boy had encephalitis and a 1-week-old male had complications from cardiorespiratory failure. Neither had typical symptoms of HFMD or HA.

**Table 3.** Subject characteristics in the second-stage survey

Variables	Non-severe^a^ *N* = 285	Severe^b^ *N* = 58
	
	*n* (%)	*n* (%)
Sex		
Male	156 (55)	39 (67)
Unknown	1	0
Years of age		
median (range)	2.8 (0–14.8)	1.7 (0–13.9)
Presence of typical symptoms of HFMD/HA		
HFMD	129 (45)	20 (34)
HA	99 (35)	5 (9)
Presence of associated complications		
Yes	108 (38)	48 (82)
Aseptic meningitis	100 (35)	33 (57)
Brainstem encephalitis	3 (1)	4 (7)
Encephalitis	5 (2)	7 (12)
Encephalomyelitis	1 (0.4)	1 (2)
Acute flaccid paralysis	0 (0)	2 (3)
Autonomic nervous system dysregulation	0 (0)	0 (0)
Pulmonary edema/hemorrhage	0 (0)	0 (0)
Cardiorespiratory failure	0 (0)	2 (3)
Underlying medical condition^c^		
Yes	59 (21)	10 (17)
Unknown	2	0
Fever ≥39°C on admission		
Yes	75 (26)	11 (19)
≥3 days from symptom onset to admission^d^		
Yes	80 (29)	21 (36)
Unknown	9	0
Leucocytosis of >17,500/mm^3^ on admission		
Yes	21 (8)	4 (7)
Unknown	5	0
Elevated AST on admission		
Yes	12 (4)	6 (11)
Unknown	16	1
Elevated ALT on admission		
Yes	12 (5)	6 (11)
Unknown	11	1
Elevated CK on admission		
Yes	0 (0)	3 (6)
Unknown	80	9
CRP ≥0.3 mg/dL on admission		
Yes	184 (66)	25 (43)
Unknown	5	0
Hyperglycemia of >8.3 mmol/dL on admission		
Yes	9 (4)	6 (11)
Unknown	69	5
Clinical outcomes		
Sequela	0 (0)	2 (3)
Died	0 (0)	2 (3)
Recovered	285 (100)	0 (0)
Pathological test		
Test conducted	61 (21)	32 (56)
Unknown	0	1
EV positive	30 (49)	10 (31)
EV71 positive	14 (23)	4 (13)

ALT, alanine aminotransferase; AST, aspartate transaminase; CK, creatine kinase; CRP, C-reactive protein; EV, enterovirus; HA, herpangina; HFMD, hand, foot and mouth disease.

^a^Non-severe cases were those with <7 days of admission.

^b^Severe cases were those with ≥7 days of admission.

^c^Any underlying medical conditions include asthma, allergy, heart disease, kidney disease, endocrine disease, neurological disease, immune suppressive condition, and other diseases.

^d^Date of symptom onset was regarded as a date when any of the following appeared: fever, rashes and/or oral ulcers, or neurological sign.

Percentages of categories in each group in parentheses were calculated excluding subjects whose variable was unknown.

**Figure 3.**
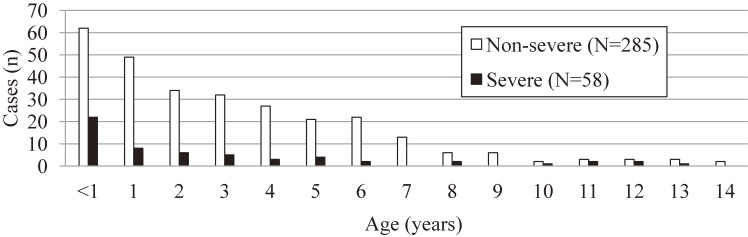
Age distribution of subjects

The peak incidence of disease for both non-severe and severe cases was in patients <1 year of age, and the incidence gradually decreased as age increased (Figure 3). Factors associated with severe cases are shown in Table 4. There was no association between severe cases and being male. Significantly increased ORs of younger age were demonstrated in multivariate analysis, but not in crude analysis. Both in model 1 and model 2, the presence of associated complications was significantly associated with severe cases. Marginal significance in increased ORs was observed for hyperglycemia in model 2.

**Table 4.** Factors associated with severe cases

Variables	Non-severe^a^ *N* = 285	Severe^b^ *N* = 58	Crude	Multivariate model 1^c^	Multivariate model 2^d^

	*n* (%)	*n* (%)	OR (95% CI)	OR (95% CI)	OR (95% CI)
Male sex					
Female	128 (45)	19 (33)	1.00	1.00	1.00
Male	156 (55)	39 (67)	1.68 (0.93 to 3.06)^*^	1.12 (0.55 to 2.28)	0.88 (0.42 to 1.85)
Unknown	1	0			
Age (years)					
≥5	81 (28)	14 (24)	1.00	1.00	1.00
3 to <5	59 (21)	8 (14)	0.79 (0.31 to 1.99)	1.39 (0.47 to 4.09)	1.61 (0.52 to 4.94)
1 to <3	83 (29)	14 (24)	0.98 (0.44 to 2.18)	2.97 (1.12 to 7.85)^**^	2.94 (1.02 to 8.51)^**^
<1	62 (22)	22 (38)	2.05 (0.97 to 4.33)^*^	5.12 (2.04 to 12.9)^**^	5.74 (2.14 to 15.4)^**^
Presence of associated complications					
No	177 (62)	10 (17)	1.00	1.00	1.00
Yes	108 (38)	48 (83)	7.87 (3.82 to 16.2)^**^	10.6 (4.45 to 25.1)^**^	7.84 (3.08 to 20.0)^**^
Any underlying medical condition					
No	224 (79)	48 (83)	1.00	1.00	1.00
Yes	59 (21)	10 (17)	0.79 (0.38 to 1.66)	0.94 (0.40 to 2.26)	0.99 (0.39 to 2.49)
Unknown	2	0			
Fever ≥39.0°C on admission					
No	210 (74)	47 (81)	1.00	1.00	1.00
Yes	75 (26)	11 (19)	0.66 (0.32 to 1.33)	0.61 (0.27 to 1.37)	0.58 (0.24 to 1.37)
Days from symptom onset to admission^e^					
<3	196 (72)	37 (64)	1.00	1.00	1.00
≥3	80 (28)	21 (36)	1.39 (0.77–2.52)	1.70 (0.81 to 3.58)	1.91 (0.85 to 4.29)
Unknown	9	0			
Leucocytosis (white blood cell count of ≥17,500/mm^3^) on admission					
No	259 (93)	54 (93)	1.00	1.00	1.00
Yes	21 (7)	4 (7)	0.91 (0.30 to 2.77)	1.44 (0.38–5.49)	0.48 (0.09 to 2.68)
Unknown	5	0			
Elevated ALT on admission					
No	261 (95)	51 (89)	1.00	1.00	1.00
Yes	13 (5)	6 (11)	2.36 (0.86 to 6.50)^*^	2.30 (0.66 to 8.06)	1.59 (0.41–6.26)
Unknown	11	1			
CRP level on admission					
<0.3 mg/dL	96 (34)	33 (57)	1.00	1.00	1.00
≥0.3 mg/dL	184 (66)	25 (43)	0.40 (0.22 to 0.70)^**^	0.65 (0.32 to 1.32)	0.58 (0.27 to 1.25)
Unknown	5	0			
Hyperglycemia (glucose level of ≥8.3 mmol/L) on admission					
No	207 (96)	47 (89)	1.00		1.00
Yes	9 (4)	6 (11)	2.94 (1.00 to 8.65)^*^		3.60 (0.94 to 13.8)^*^
Unknown	69	5			

ALT, alanine aminotransferase; CI, confidence interval; CRP, C-reactive protein; OR, odds ratio.

^a^Non-severe cases were those with <7 days of admission.

^b^Severe cases were those with ≥7 days of admission.

^c^Model included all variables in the table except for hyperglycemia. Analyses were based on 262 non-severe and 57 severe patients without missing explanatory variables. Hosmer-Lemeshow test, *P* = 0.270.

^d^Model included all variables in the table. Analyses were based on 206 non-severe and 52 severe patients without missing explanatory variables.

Hosmer-Lemeshow test, *P* = 0.430.

^e^Date of symptom onset was regarded as a date when any of the following appeared: fever, rashes and/or oral ulcers, or neurological sign.

^*^P < 0.10 ^**^P < 0.05

Percentages of categories in each group in parentheses were calculated excluding subjects whose variable was unknown.

## DISCUSSION

Our study estimated the number of pediatric inpatients with clinically defined HFMD, HA, or associated complications during an epidemic period with predominant EV71 infections in 2010. Factors associated with severity were also explored. In Japan, there has been one nationwide survey with admitted cases of HFMD between 2000 and 2002,^30^ in which risk factors of severe cases were explored but the number of patients was not estimated. The present study is the first to reveal disease burden of HFMD, HA, and associated complications in Japan by estimating inpatients during an epidemic period. We also examined laboratory test results, not only basic demographic characteristics.

The estimated number of inpatients and the cumulative incidence of hospitalized cases among children <15 years of age during the 6-month epidemic period were calculated as approximately 2,900 and 17.0 per 100,000 population, respectively. Case-fatality proportion in our study was 0.57% (2/352), which may be higher than that of a total year since our observation was limited to the epidemic period. Nevertheless, this proportion was much lower than that of the 1998 outbreak in Taiwan (19.3%)^2^ and the Chinese 2008–2012 surveillance data (3.0%).^36^ This could be a result of the difference in accessibility to medical care, virulence of different genogroups/sub-genogroups of EV71,^37^^,^^38^ population immunity to EV71, or other miscellaneous host factors.

Regarding factors associated with severe cases, the OR for male sex did not increase, but the ORs for younger ages did increase in multivariate analysis. Some studies using observational or univariate analyses have claimed male predominance among severe patients.^14^^,^^33^^,^^39^ Other studies looking at subjects with male predominance suggested that younger age was a risk factor according to multivariate analysis.^36^^,^^40^ In our study subjects, age rather than sex was related to severity of HFMD, HA, and associated complications.

The increased OR for presence of associated complications was an expected finding. Although we also examined whether duration of ≥3 days from symptom onset to admission was associated with severe cases, elevated ORs of 1.70 (model 1) and 1.91 (model 2) were not statistically significant. Delayed hospital visit was found to be a risk factor for complicated EV71 infection in a Taiwan study.^34^ Some studies have also indicated that longer duration of fever was an independent risk factor.^9^^,^^33^^,^^41^ It is possible that limited access to medical institutions led to longer duration of time from symptom onset to admission. If a certain amount of time passes after a child develops symptoms, including fever, rashes, or neurological symptoms, their condition may deteriorate and careful observation may be required. Further studies with more Japanese subjects are required to shed light on this issue.

Hyperglycemia was likely to be an important laboratory indicator for severe cases in the present study, which is in line with findings from previous studies that demonstrated higher mean glucose levels or increased proportions of hyperglycemia in severe HFMD cases that included pulmonary edema^33^^,^^42^^,^^43^; however, there were no cases of pulmonary edema in our study. It is possible that systemic sepsis might have induced the state of hyperglycemia in those patients because alteration in hepatic metabolism and insulin resistance was stimulated in response to viral infection.^2^^,^^44^^,^^45^ It has also been suggested that stimulation of the sympathetic nervous system might induce the loss of blood glucose homeostasis.^33^ On the other hand, it is possible that non-severe cases with missing values of blood glucose levels in our study had hyperglycemia. If so, our finding of increased OR for hyperglycemia may be overestimated. However, ORs for other variables in model 1 did not meaningfully differ from those in model 2, in which 85 patients were not included, mainly due to missing values for hyperglycemia, suggesting that overestimation may not have occurred.

One strength of our study is that it was a nationwide survey with a high response proportion (approximately 70%). Our study was the first to estimate the number of inpatients with HFMD, HA, and associated complications in Japan. In addition, it is noteworthy that the study observation period covered the time during which an HFMD epidemic was caused by EV71 infection and greater attention was being paid to public health. Adoption of the WPRO definitions was another unique characteristic of this study. To our knowledge, previously published studies used their own definitions and, therefore, were not comparable to one another. Our study results will be comparable to future similar studies that adopt the WPRO definitions.

There were several limitations to the present study. First, our estimates were based on the assumption that, if the epidemic is homogeneous across the region, hospitalized rates are similar within each stratum regardless of regional variations in population density. Prefectural distribution of reported cases in the second survey (*n* = 352) did not align with that of the population under 15 years of age, whereas prefectural distribution of number of hospital pediatric departments in Japan and number of departments sampled in this survey did align. As noted earlier, EV71 was largely isolated in the western part of Japan in 2010.^25^ Although such differences in regional epidemics of EV71 might have affected unparalleled prefectural distribution of our cases, we cannot deny the possibility of selection bias and lack of representativeness. Second, we could have underestimated the number of inpatients. Pediatricians might not have reported some eligible patients in the second survey, despite our request that they not report only patients who met the exclusion criteria. Third, this retrospective study design that relied on information from pre-existing medical records resulted in a number of missing values. A very limited number of subjects had laboratory test results for enterovirus infection (93/343, 27%) because routine medical examinations did not necessarily employ laboratory diagnoses for HFMD or HA. Similarly, data on CK levels were missing in 89 patients, of whom a majority were non-severe cases. This could have resulted from pediatricians’ judgements that cases were not as severe as they did not require monitoring of these laboratory findings. Elevated CK level has been suggested as a risk factor for severe HFMD, compared to cases without complications.^40^ If CK level was recorded thoroughly and included in our analysis, we might have been able to present additional insight on association between elevated CK level and severe cases. Finally, although our observation was confined to the epidemic period of HFMD and HA, study subjects may have included those infected with agents other than enterovirus. To examine the extent of the influence of disease misclassification, we limited subjects to those with typical symptoms of HFMD or HA, and/or who had EV positive results (*n* = 274), and we additionally analyzed factors associated with severity, obtaining similar findings.

In conclusion, EV71, which has been suggestively related to severe complications, was predominant in Japan in 2010, and this study demonstrated the impact of HFMD, HA, and associated complications during an epidemic that same year. In addition to estimating the number of pediatric inpatients to have been 2,900 between April 1 and September 30, 2010, factors associated with disease severity were explored. Our findings suggested that careful observations of infected younger children, especially infants, are essential during EV71 infection epidemics. We believe that our results contribute fundamental epidemiologic evidence for HFMD, HA, and associated complications in Japan and Asia.
